# Sequential Multiple Assignment Randomized Trial (SMART) to identify optimal sequences of telemedicine interventions for improving initiation of insulin therapy: A simulation study

**DOI:** 10.1186/s12874-021-01395-7

**Published:** 2021-09-30

**Authors:** Xiaoxi Yan, David B. Matchar, Nirmali Sivapragasam, John P. Ansah, Aastha Goel, Bibhas Chakraborty

**Affiliations:** 1 Centre for Quantitative Medicine. Duke-NUS Medical School, 8 College Road, Singapore, 169857 Singapore; 2 Health Services and Systems Research Department, Duke-NUS Medical School, 8 College Road, Singapore, 169857 Singapore; 3 Department of Medicine, Duke University Medical Center, Durham, North Carolina USA; 4 Department of Statistics and Applied Probability, Faculty of Science, National University of Singapore, Singapore, 117546 Singapore; 5 Department of Biostatistics and Bioinformatics, Duke University, Durham, North Carolina USA

**Keywords:** Sequential treatment designs, telemedicine, diabetes, optimal adaptive interventions

## Abstract

**Background:**

To examine the value of a Sequential Multiple Assignment Randomized Trial (SMART) design compared to a conventional randomized control trial (RCT) for telemedicine strategies to support titration of insulin therapy for Type 2 Diabetes Mellitus (T2DM) patients new to insulin.

**Methods:**

Microsimulation models were created in R using a synthetic sample based on primary data from 63 subjects enrolled in a pilot study of a smartphone application (App), *Diabetes Pal* compared to a nurse-based telemedicine strategy (Nurse). For comparability, the SMART and an RCT design were constructed to allow comparison of four (embedded) adaptive interventions (AIs).

**Results:**

In the base case scenario, the SMART has similar overall mean expected HbA1c and cost per subject compared with RCT, for sample size of *n =* 100 over 10,000 simulations. SMART has lower (better) standard deviations of the mean expected HbA1c per AI, and higher efficiency of choosing the correct AI across various sample sizes. The differences between SMART and RCT become apparent as sample size decreases. For both trial designs, the threshold value at which a subject was deemed to have been responsive at an intermediate point in the trial had an optimal choice (i.e., the sensitivity curve had a U-shape). SMART design dominates the RCT, in the overall mean HbA1c (lower value) when the threshold value is close to optimal.

**Conclusions:**

SMART is suited to evaluating the efficacy of different sequences of treatment options, in addition to the advantage of providing information on optimal treatment sequences.

**Supplementary Information:**

The online version contains supplementary material available at 10.1186/s12874-021-01395-7.

## Introduction

A major objective of clinical trials, particularly randomized controlled trials (RCTs) is to identify which of two or more therapies is most effective. However, people often differ in their response to the same intervention. When a treatment that works for most people based on an RCT is not effective for a particular patient, in clinical practice the next step typically is to try something else. The next choice in this “trial and error” process would, ideally, be informed by evidence. However, clinical trials in which individuals are randomized to sequences of treatment strategies are seldom used [1].

An alternative to an idiosyncratic series of choices are decision rules such as those embodied in guidelines developed by medical professional organizations: a combination of expert opinion, behavioral, psychosocial and biological theories, and observational studies to formulate adaptive treatment algorithms, or adaptive interventions (AIs) [2, 3]. While clinical guidelines may reduce variability from practice to practice, they do not alleviate the scientific uncertainty about which sequence is actually optimal. The recommendations become the subject of potential future research.

Experimental trial designs have been proposed for development and optimization of treatment sequences. One such design is the Sequential Multiple Assignment Randomized Trial (SMART) [2, 4]. Adaptive interventions are treatment algorithms wherein treatment is sequentially modified over time based on individual’s response. The rationale is that by adjusting the treatment type and level as a function of time-dependent measures such as response to the past treatment, the long-term outcome is optimized [2, 5].

Most experience with SMARTs has been limited to mental health and behavioral sciences [2, 4], and Phase 2 trials in oncology [6]. SMART is particularly attractive in cancer therapy as sequential treatment based on intermediate response is already well-established. However, SMART has potential value to scientifically address problems in a wide range of contexts, including the use of technology such as telemedicine to encourage health-promoting behaviors [7].

Telemedicine is the provision of healthcare services and the exchange of healthcare information using information and communication technology across distances [8, 9]. It is used in multiple areas of clinical practice, e.g., surgical practices [10–12], management of chronic diseases [13], addiction management [14] and palliative care [15, 16]. The necessity for and utilization of telemedicine has significantly accelerated, when many in-person clinical activities are deferred or suspended, as a result of the on-going coronavirus disease of 2019 (Covid-19) pandemic [17, 18]. What is becoming evident in this field is that “one size does not fit all”. Studies have shown that telemedicine interventions are more likely to have a positive effect on users’ self-efficacy, knowledge relevant to their condition, and behavioral and clinical outcomes [19]. However, not all patients are receptive to a particular mode of delivery. A key to establishing the effective and cost-effective application of telemedicine is understanding how these approaches fit into real-world care, in particular as part of a sequence that maximizes the proportion of patients who ultimately respond to good effect.

With this in mind, we sought to examine the value of a SMART design compared to an RCT for two telemedicine strategies to support titration of insulin therapy for Type 2 Diabetes Mellitus (T2DM) patients new to insulin: (1) a largely self-contained smartphone app, Diabetes Pal [20] and (2) a nurse-based telephone consultation service, SingHealth Polyclinics’ (SHP) Insulin Initiation Telecare Program (see the Methods section for details about these two telemedicine modalities). For comparability, the SMART and an RCT designs were constructed to allow comparison of various sequences of the two telemedicine strategies. The basis for this comparison is microsimulation using data derived from a pilot clinical trial of Diabetes Pal [20]. We sought to demonstrate the impact of the two trial designs on improvement in chronic blood glucose control as measured by change in glycated hemoglobin (HbA1c), and trial cost for the study population. In sensitivity analysis we examined how these measures of value were affected by various aspects of trial design, including the operating characteristics of the measure of responsiveness to initial treatment measure used to determine whether to continue or switch treatment.

## Methods

### Overview of the Simulation Study

The purpose of our simulation study was to conduct a head-to-head comparison between two design approaches intended to identify the optimal sequence of the two telemedicine modalities for titration of insulin dose in insulin-naïve diabetic patients. Although it is impractical to compare the design approaches directly using the same set of participants in real-life empirical studies, such comparisons are possible in a computer simulation. Specifically, for this study we developed a microsimulation created in R 3.6.1 [21]. The synthetic subjects were generated based on the characteristics of the real subjects in the pilot study of the *Diabetes Pal* app [20].

### Two Telemedicine Intervention Modalities

Here we briefly describe the telemedicine intervention modalities that were compared in the pilot study, and informed by that, were considered in our simulation study.

**Fig. 1:**
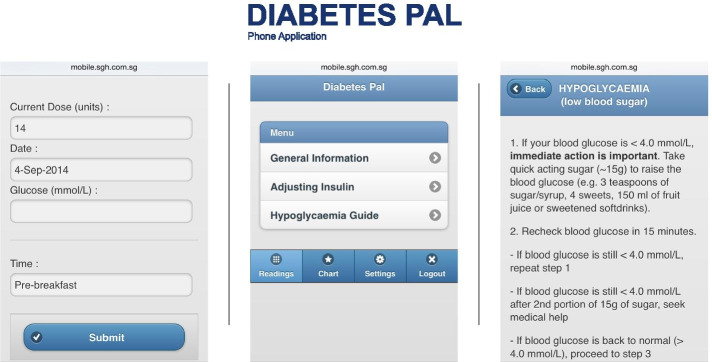
Diabetes pal. From "Diabetes Pal" by Integrated Health Information Systems (https://www.ihis.com.sg/Project_Showcase/Mobile_Applications/Pages/Diabetes_Pal.aspx). Copyright 2021 by Integrated Health Information Systems (IHIS) Pte Ltd

- *SingHealth Polyclinics’ Insulin Initiation Telecare Program (Nurse)*: The program was designed to support insulin initiation for patients with T2DM at the primary care practices of SingHealth, the largest public healthcare group in Singapore. Designated primary care nurses were trained as care managers, to assist patients with insulin initiation via weekly telephone consultations. These consultations included checking of current insulin dose and presence of symptoms of hypoglycemia, and titration of next insulin dose. Throughout this article, we will refer to this telemedicine intervention as ‘*Nurse*’.
- *Smartphone Application based Telecare Program (App)*: Under this program, self-titration using the smartphone app *Diabetes Pal* [20] (Fig. 1) with minimal telephone-based support from care managers for insulin initiation, was proposed. Diabetes Pal is a smartphone application that allows a diabetic patient to self-titrate their insulin doses. Patient self-titration of insulin dose based on a prescribed algorithm has been shown to be safe and efficacious in improving glycemic control [22]. The app was developed by Integrated Health Information Systems Ltd. (IHiS) and has been tested for its feasibility to deliver the insulin titration algorithm in insulin-naïve patients in a pilot study recently conducted at the Singapore General Hospital [20]. Throughout this article, we will refer to this telemedicine intervention as ‘*App*’.

### Operationalization of Competing Trial Designs

To compare a traditional RCT and SMART for evaluation of effectiveness and cost in the trial context, we implemented a microsimulation of these two trial designs run over a 12-week “study” period.

- *SMART Design*

The SMART design operates in two stages. At stage one, all patients were randomized with a 1:1 ratio between Nurse and App. However, at the end of stage 1 (6 weeks from the initial randomization), patients were categorized as either a responder (R = 1) or a non-responder (R = 0) based on their reduction in HbA1c value in the 6-week period. Based on evidence in literature [23], insulin therapy is rapidly effective and known to reduce HbA1c levels in the range of 1.5 to 3.5 (conditional on other baseline values). In the pilot study data, the mean reduction in HbA1c after 6 weeks was 0.92 (SD = 0.71). Considering these pieces of information and clinical expert inputs, in the base-case scenario, the threshold for declaring a response was assumed to be 0.5% (i.e., the patient was considered a responder if the reduction in HbA1c from baseline is ≥0.5%, and a non-responder otherwise.).

**Fig. 2:**
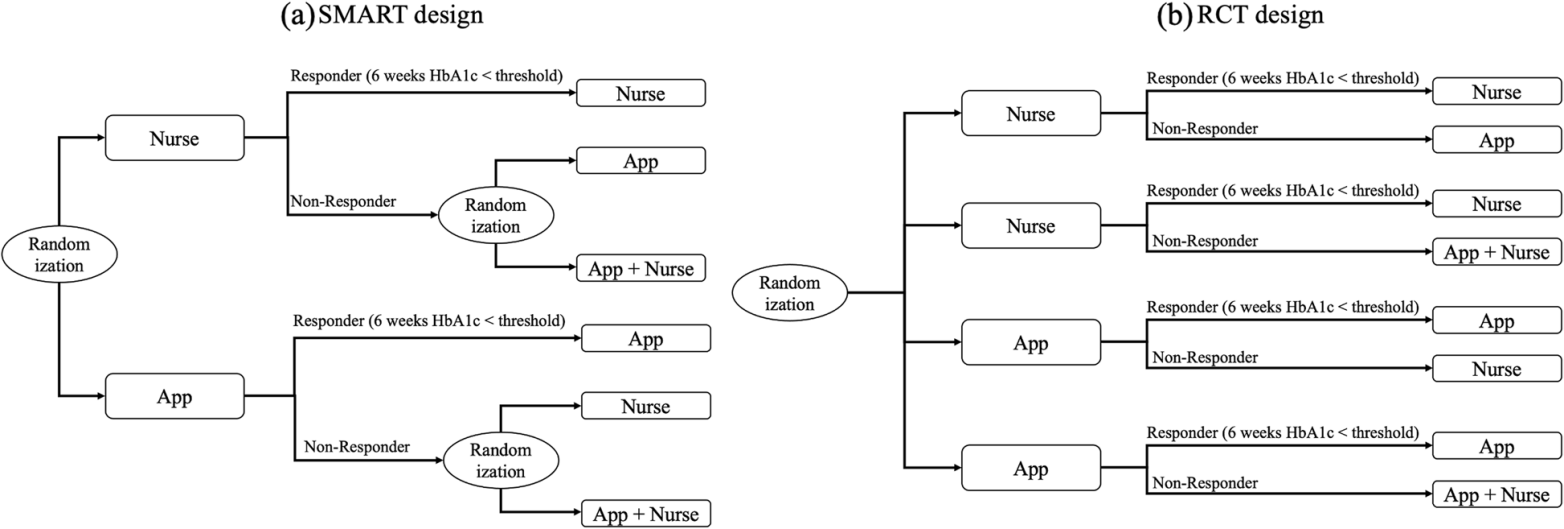
The (a) SMART and (b) RCT design in comparison. Each intervention component (Nurse, App, App + Nurse) is for 6 weeks long. (a) Randomisation happens at baseline for stage 1 intervention and at week 6 for stage 2 intervention, given stage 1 treatment and response. The four AIs are embedded in the design as a result. (b) Randomisation happens at baseline only, so the four arms correspond to the four AIs

The value was also varied in sensitivity analysis. The responders to the first-stage intervention continued with the same intervention in stage 2 (weeks 6–12 of the study). However, the non-responders were re-randomized to either a switch to the intervention not tried before for the same patient or a combined intervention (App + Nurse). The non-responders were re-randomized in a 1:1 ratio between the switch option and the combination option. A schematic of the SMART design is presented in Fig. 2 (a). Because of the re-randomization at stage 2, the current SMART design offers a comparison between four embedded adaptive interventions (AIs) (see [24]), described in Table 1.

**Table 1:** Description of the four adaptive interventions (AI). N = Nurse, A = App and A + N = App + Nurse

AI#	Notation	Description
AI1	(*N*, *N*^*R*^*A*^1 − *R*^)	Nurse at stage 1, continue with Nurse at stage 2 if responder, or switch to App at stage 2 if non-responder.
AI2	(*N*, *N*^*R*^(*A* + *N*)^1 − *R*^)	Nurse at stage 1, continue with Nurse at stage 2 if responder, or provide combination at stage 2 if non-responder.
AI3	(*A*, *A*^*R*^*N*^1 − *R*^)	App at stage 1, continue with App at stage 2 if responder, or switch to Nurse at stage 2 if non-responder.
AI4	(*A*, *A*^*R*^(*A* + *N*)^1 − *R*^)	App at stage 1, continue with App at stage 2 if responder, or provide combination at stage 2 if non-responder.

The primary outcome (Y) was the HbA1c measurement at the end of the trial, and was recorded for all patients. The expected outcomes corresponding to the four embedded adaptive interventions are denoted *E*(*Y*)_*AI*1_, *E*(*Y*)_*AI*2_, *E*(*Y*)_*AI*3_, and *E*(*Y*)_*AI*4_. Also, the average outcome corresponding to the best AI from the SMART is denoted by min[*E*(*Y*)_*AI*1_, *E*(*Y*)_*AI*2_, *E*(*Y*)_*AI*3_, *E*(*Y*)_*AI*4_] and the best AI being $\arg \underset{j\in \left\{1,..,4\right\}}{\ \min }E{(Y)}_{AIj}$; this is a key performance metric of the SMART design that we compare with the best outcome from the RCT design, in our simulation study. Note that for SMART, *E*(*Y*)_*AI*1_, *E*(*Y*)_*AI*2_, E(*Y*)_*AI*3_, and *E*(*Y*)_*AI*4_ need to be estimated using the inverse probability weighting method [25].

(b) *RCT Design*

The RCT design (Fig. 2 (b)) is a conventional randomization designed to test the same sequences of treatments as the SMART design with the same intermediate evaluation at 6-weeks for responsiveness to initial treatment. However, for individuals deemed not to respond to the initial treatment, the new treatment was established at the time of initial randomization rather than at 6-weeks. In other words, the four AIs are separate arms in the design, where individuals are assigned to one of the AI arms at the start of the trial. This is different from the SMART design, where the AIs are embedded. As with SMART, effectiveness and cost are assessed based on final HbA1c and cost at the trial end at 12 weeks.

### Data Generation Model

#### Baseline HbA1c

The pilot study [20] of the Diabetes Pal smartphone app enrolled 66 insulin naïve patients with suboptimal glycemic control of HbA1c ≥ 7.5% despite use of 2 or more oral glucose lowering drugs. These patients were between 30 and 70 years of age. Of these 66 recruited subjects, only 63 patients had complete follow-up data. Based on the baseline HbA1c measurements of these 63 patients, a normal distribution with mean = 9.73 and SD = 1.37 was used to generate the baseline HbA1c values (*Y*_0_) of the hypothetical patients in the simulation model. Detailed description of the model generation and algorithm may be found in the Additional file 1.

#### Receptiveness

In addition to the starting level of glycemic control measured by HbA1c, two additional subject characteristics were assigned at baseline: (1) receptiveness to the App (*Rc*_*A*_), and (2) receptiveness to the Nurse (*Rc*_*N*_). A patient is deemed “receptive” if their engagement with the specific intervention had more than a nominal impact on their tendency to improve glycemic control. This was operationalized as a binary indicator variable (1 = receptive, 0 = non-receptive). Since information on receptiveness was not collected during the pilot study, estimates from the literature were utilized. Specifically, according to Deloitte’s Global Mobile Consumer Survey 2016 for UK [26], 69% of the smartphone users made standard voice calls weekly. It was assumed that the smartphone users who made standard voice calls were comfortable communicating over telephone and therefore would be receptive to receiving insulin titration information over telephone via the Nurse. The survey also reported that around 51% of the users downloaded more than five apps on their smartphones. Supported by the data in the survey, it was assumed that the users who downloaded more than five apps would be using the apps to carry out activities (other than to communicate) that involved inputting and outputting of information; it was further assumed that these people would also be receptive to using App to carry out insulin titration. We further assumed that the receptiveness to one intervention was independent of the receptiveness to the other intervention. Thus, in our microsimulation study the tendency for *Rc*_*A*_ and *Rc*_*N*_ were based on the probability of success in Bernoulli trials (receptiveness rates) 0.51 and 0.69, respectively. Furthermore, we assumed that subjects who were receptive to at least one of the two interventions were likely to be receptive to the combined intervention (App + Nurse), which is only given at stage 2 (see Fig. 2). The average probability of receptiveness to the combination, assuming they were initially not receptive to the initial intervention, was calculated to be 0.75 (for detailed calculation, refer to Model Development in Additional file 1).

#### Change in HbA1c conditional on being receptive to a received intervention

In the microsimulation, the change in HbA1c for each individual was drawn from a normal distribution corresponding to whether the subject was actually receptive or not (as assigned at baseline). As noted, receptiveness to an intervention, leading to appropriate changes in insulin dose, was assumed to be reflected in improvement in HbA1c over a 6-week period beyond random change. To generate plausible HbA1c change distributions, we stratified data from the actual trial subjects by 6-week change in HbA1c and calculated the means and standard deviations. The mean HbA1c reductions at weeks 6 and 12 were 0.92 (SD = 0.71) and 0.56 (SD = 0.77). To separate these values for receptive and non-receptive participants, we assumed an average receptiveness rate of 60% (e.g., by averaging the receptiveness rates to App and Nurse) in the trial subjects and fixed the mean for non-receptive participants to be zero (i.e., HbA1c reductions at week 6 and 12 for non-receptive participants are assumed to be 0 (SD = 0.71) and 0 (SD = 0.77)). Thus, the mean HbA1c reductions at weeks 6 and 12 for receptive participants are assumed to be 1.53 (SD = 0.71) and 0.94 (SD =0.77), respectively.

#### Calculation of Trial Costs

In the microsimulation, costs were accumulated for each synthetic subject based on their interventions experienced, including the time costs of providing the interventions, monitoring, and re-randomization when needed. The time and cost components (see Tables A1 and A2 of Additional file 1) were based on the actual expenditures incurred during the pilot study and expert inputs. Because costs for the trial are almost entirely personnel time, we did not include cost of the app itself. Cost was calculated in US dollars (USD) by multiplying personnel time by the exchange rate adjusted modal wage rate for Singapore, a country with a gross domestic product per capita comparable to the US, approximately USD 57,000. Based on these, the accumulated cost for each synthetic subject ranges from USD 312.55 to USD 382.00.

#### Analysis

In our simulation study, we performed a base case analysis in which the two designs were compared with the key input parameters fixed as follows:

- receptiveness rate to the Nurse (*P*(*Rc*_*N*_ = 1)) = 69%,
- receptiveness rate to the App (*P*(*Rc*_*A*_ = 1)) = 51%,
- receptiveness rate to the App + Nurse (*P*(*Rc*_*A* + *N*_ = 1)) = 75%,
- reduction threshold for response (*δ*) = 0.5%, and
- trial size of patient cohort (*n*) = 100.

The Monte Carlo assessments were based on simulation size (B) = 10,000. For each design, the Monte Carlo mean HbA1c estimate ($\overset{\sim }{y}=\frac{\sum_{b=1}^B{\overline{y}}_b}{B}\Big)$ and the standard deviation $\left({s}_{\overset{\sim }{y}}=\sqrt{\frac{\sum_{b=1}^B{\left({\overline{y}}_{b-}\overset{\sim }{y}\right)}^2\ }{\left(B-1\right)}}\right)$ were calculated where ${\overline{y}}_b$ is the overall and per AI mean HbA1c for the *b*^*th*^ simulated trial. The probability of selecting the best AI as defined in *SMART design* over B simulations was calculated. In sensitivity analysis, we varied each parameter over a broad range to determine if there was any change in the sign for the difference in effectiveness or cost, large absolute changes on outcomes for both designs, or non-linear relationships between the parameters and outcomes.

## Results

### Base case

**Fig. 3:**
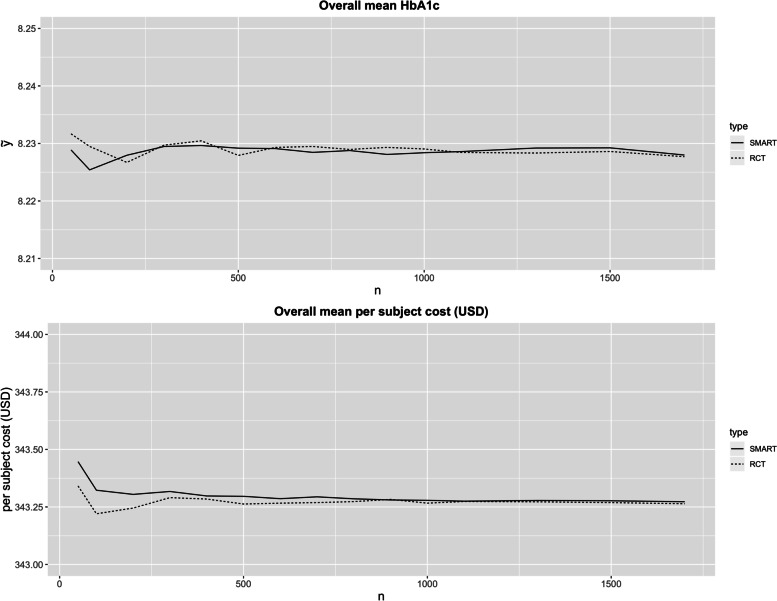
The Monte Carlo means of the final outcome HbA1c and per subject cost (USD) for different sample sizes

**Fig. 4:**
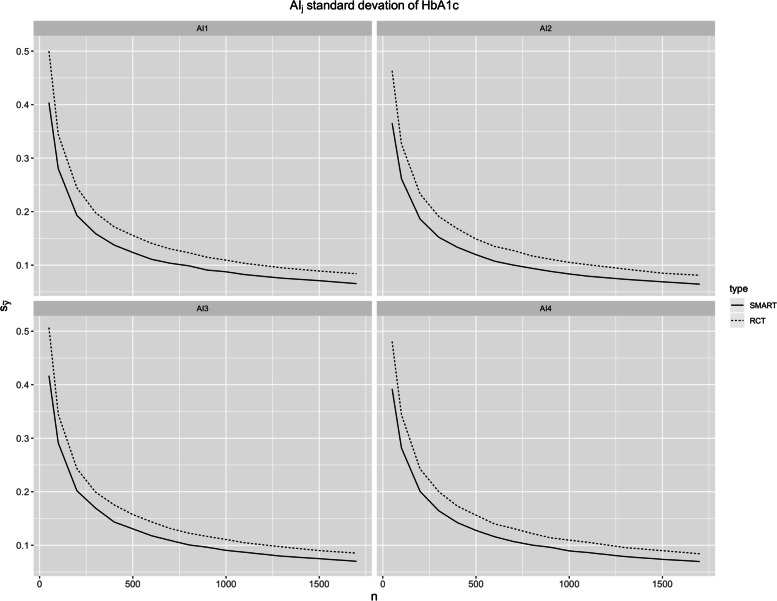
The standard deviations of the Monte Carlo means of the final outcome HbA1c by AIs for different sample sizes

**Fig. 5:**
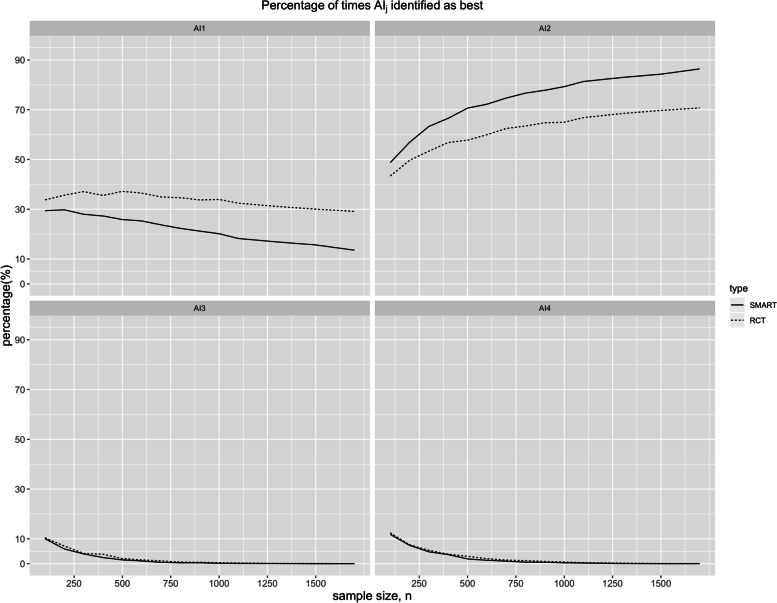
The number of times out of B = 10,000 simulations, the SMART and RCT trial identify the j-th AI (j = 1, … , 4) as the best AI across a wide range of sample sizes. According to the data generation model of the simulation study, AI2 is the truly optimal AI

In the base case scenario (Table 2), the difference of the overall mean expected HbA1c and per subject cost between the SMART and the RCT design is almost negligible (both HbA1c are 8.28%; per subject cost is USD 343.32 vs USD 343.22). As the sample size increases, the overall effectiveness and cost between SMART and RCT designs are essentially equivalent (Fig. 3). This is expected since both designs are unbiased for estimating the above metrics. Although the estimated mean HbA1c by AIs are similar in both designs, the standard deviations are noticeably lower in case of the SMART design. This is true across a wide range of sample sizes, approaching zero as trial size became very large (Fig. 4). Given the underlying data generation (see Additional file 1), the true optimal AI is AI2. The SMART design is able to outperform the RCT design by having higher probability of choosing AI2 given same sample size *n =* 100 (48.73% vs 43.38%). The efficiency of the SMART design becomes apparent as we aim to maximize the probability of choosing AI2 by varying the sample sizes (Fig. 5). In order to have approximately 70% probability of correctly choosing AI2, SMART requires *n =* 500 (70.66%), whereas RCT requires *n =* 1700 (70.72%).

**Table 2:** The Monte Carlo mean (standard deviation) of the final HbA1c outcomes (Y) and cost per subject and the probability of selecting each AI as optimal (p_optAI_) for SMART and RCT of sample size *n =* 100. The simulation size was 10,000. According to the data generation model of the simulation study, AI2 is the truly optimal AI

	SMART	RCT
**Overall**		
Y	8.23 (0.17)	8.23 (0.17)
Cost (USD)	343.32 (2.14)	343.22 (2.16)
**By (embedded) AI**		
Y_AI1_	8.12 (0.28)	8.12 (0.34)
Y_AI2_	8.04 (0.26)	8.05 (0.33)
Y_AI3_	8.39 (0.29)	8.39 (0.34)
Y_AI4_	8.36 (0.28)	8.36 (0.34)
p_optAI1_	29.41%	33.77%
p_optAI2_	48.73%	43.38%
p_optAI3_	10.06%	10.43%
p_optAI4_	11.80%	12.42%

### Sensitivity analyses

**Fig. 6:**
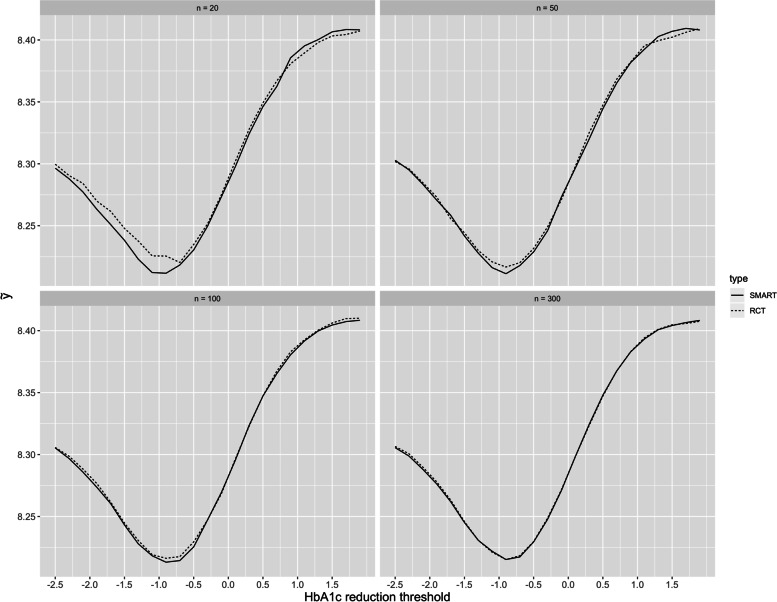
The overall mean expected HbA1c from 10,000 simulated SMART and RCT trials for sample size *n =* 20, 50, 100, 300 varied across a wide range of reduction threshold values

For both trial designs the threshold value at which a subject was deemed to have been responsive at an intermediate point in the trial had an optimal value (i.e., the sensitivity curve had a U-shape) (Fig. 6). As the threshold moves away in either direction from the optimal value, the mean HbA1c for the trial subjects worsens. Under the optimal threshold, the SMART design becomes more efficient than the RCT design when the sample size is small, because the final overall mean HbA1c performs better (that is, it has a lower value). This change in threshold corresponds to a change in the relationship between the sensitivity and specificity of the interim evaluation of responsiveness. A more negative threshold results in lower sensitivity but greater specificity for responders while a higher threshold results in higher sensitivity but lower specificity.

## Discussion

In this study, we examined the value of the SMART design relative to a comparable RCT design of two telemedicine interventions for insulin initiation: a largely self-contained smartphone app [20] and a nurse-based telephone consultation service. The designs were comparable in that both had the aim to evaluate the optimal sequencing of these two interventions, including the potential for combining interventions. We did this evaluation using microsimulation drawing on empirical data from a prior conventional trial. Simulation allowed us to perform sensitivity analysis of how diabetes control (as assessed by HbA1c) and trial costs were impacted by various aspects of trial design, including the operating characteristics of the intermediate measure used in the SMART and RCT designs to continue or switch treatment. It should be noted that the RCT design used as the comparator was unconventional, involving both multiple arms and treatment switching based on interim assessment of responsiveness to the initial treatment.

While both designs provide information on the optimal sequencing of therapies, we demonstrated some notable benefits of SMART compared to RCT. First, the SMART design from the perspective of trial population, had consistently smaller variance in the mean HbA1c per AI, which was especially evident at smaller sample sizes, at approximately equivalent cost. For the same sample size, the SMART design has higher probability of identifying the best AI.

Another advantage of SMART is that the design offers the potential to personalize treatment sequences by evaluating features predictive of responsiveness by treatment order. In our present simulation study, this feature of SMART was not examined as subjects were simulated as identical with regard to all features except for responsiveness to one intervention or the other. However, there is a sizable statistical literature that offers methodologies (e.g., Q-learning) for doing such personalization as secondary analysis of SMART data [5, 27]. This aspect can be pursued in simulations as an important future work.

In sensitivity analysis, the observed benefits were robust. However, we did note that the value of both designs depended on the threshold value for defining response to treatment at the end of first stage. Average HbA1c control for trial subjects was optimal at an intermediate threshold value: too low and subjects who were unresponsive to their initial treatment were incorrectly maintained on an ineffective therapy; too high and subjects who were responsive to initial therapy would be incorrectly switched from an effective therapy. This suggests that the sensitivity and specificity of the threshold value can be important parameters to consider in SMART design and that the value of the design can be much diminished if the first stage evaluation does not have good operating characteristics.

Most clinical trials aim to conduct formal hypothesis tests in order to determine the superior interventions. However, in case of telemedicine, it may often be of more interest to find out if a cheaper or less burdensome intervention (e.g. App) is non-inferior to an established but more expensive intervention (e.g. Nurse). Such non-inferiority testing methodologies have been applied to conventional RCTs for many years [28]. Very recently, such non-inferiority testing methods [29] along with free web-based software [30], have also been developed in the SMART design context. Availability of such methodology and software tools brings SMARTs to an even playing field as RCTs, in terms of flexibility of hypothesis testing and data analysis. We have not considered non-inferiority testing in the current manuscript.

The primary goal of SMART is to learn – through *within-patient adaptation* of interventions over stages – an optimal strategy that can benefit future patients beyond the trial, not the trial participants *per se*. As such, it does not allow *between-patient adaptation* of interventions within the trial, because the randomization probabilities in a SMART are pre-specified. This fixed allocation scheme in a SMART design (as in conventional RCT) is motivated by the aim to maximize statistical power in order to maximize the scientific information gained from the trial. However, there are settings (e.g., implementation studies) where there is urgent need to translate emerging evidence from ongoing trials into practice, including the remainder of the trial participants, in order to maximize the benefit to the overall population of interest [20]. This need can be accommodated in both a SMART and an RCT through the machinery of response-adaptive allocation. Such an adaptive SMART or adaptive RCT design would allow modification of the randomization probabilities based on observed outcome data, favoring the treatment sequences that empirically look better (even though not statistically significant), at pre-set interim times during the trial [6, 31, 32]. For simplicity, we chose not to consider such a response-adaptive SMART or RCT in our current simulation study. However, we feel that such designs can potentially be even more attractive in the telemedicine context, optimizing welfare of trial participants while also finding optimal care strategies for future patients. We view more in-depth study of such designs in the telemedicine arena as an important future work.

## Conclusion

In light of increasingly complex care management questions, new trial designs have been offered to improve the range of useful inferences that can be derived from clinical trials. SMART is one example that is particularly suited to evaluating the efficacy of different sequences of treatment options. To make better use of SMART, it is important to understand the advantages and disadvantages of SMART relative to a conventional design. This study illustrates the advantages of the SMART design over a comparable RCT for evaluating sequences of therapies. We note that the value of a SMART depends on the accuracy of the intermediate measure of responsiveness as well as the burden and cost of re-randomization.

## Supplementary Information

**Additional file 1.** Details of data generation model and simulation algorithm.

## Data Availability

Relevant code for the simulated data and results is available at https://github.com/xiaoxi-yan/smart-rct-comparison.

## Abbreviations

SMART: Sequential Multiple Assignment Randomized Trial
RCT: Randomized Controlled Trial
AI: Adaptive Interventions
A: App, a smartphone application, *Diabetes Pal*
N: Nurse, a nurse-based telemedicine strategy

## References

[1] Lei H (2012). A "SMART" design for building individualized treatment sequences. Annu Rev Clin Psychol.

[2] Murphy SA (2005). An experimental design for the development of adaptive treatment strategies. Stat Med.

[3] 2017 ACC/AHA/AAPA/ABC/ACPM/AGS/APhA/ASH/ASPC/NMA/PCNA (2018). Guideline for the Prevention, Detection, Evaluation, and Management of High Blood Pressure in Adults: A Report of the American College of Cardiology/American Heart Association Task Force on Clinical Practice Guidelines. J Am Coll Cardiol.

[4] Lavori PW, Dawson R (2008). Adaptive Treatment Strategies in Chronic Disease. Annu Rev Med.

[5] Chakraborty B, Moodie EEM (2013). Statistical methods for dynamic treatment regimes: reinforcement learning, causal inference, and personalized medicine.

[6] Thall PF, Sung H-G, Estey EH (2002). Selecting Therapeutic Strategies Based on Efficacy and Death in Multicourse Clinical Trials. J Am Stat Assoc.

[7] Kumar S (2013). Mobile health technology evaluation: the mHealth evidence workshop. Am J Prev Med.

[8] Craig J, Patterson V (2005). Introduction to the practice of telemedicine. J Telemed Telecare.

[9] Sood S (2007). What is telemedicine? A collection of 104 peer-reviewed perspectives and theoretical underpinnings. Telemed J E Health.

[10] Gallo G (2021). E-consensus on telemedicine in proctology: A RAND/UCLA-modified study. Surgery.

[11] Huang EY (2019). Telemedicine and telementoring in the surgical specialties: a narrative review. Am J Surg.

[12] Eadie L, Seifalian A, Davidson a (2003). Telemedicine in surgery. J Br Surg.

[13] Wootton R (2012). Twenty years of telemedicine in chronic disease management–an evidence synthesis. J Telemed Telecare.

[14] Molfenter T (2015). Trends in telemedicine use in addiction treatment. Addiction Sci Clin Pract.

[15] Worster B, Swartz K (2017). Telemedicine and palliative care: an increasing role in supportive oncology. Curr Oncol Rep.

[16] Sirintrapun SJ, Lopez AM (2018). Telemedicine in cancer care. Am Soc Clin Oncol Educ Book.

[17] Contreras CM (2020). Telemedicine: patient-provider clinical engagement during the COVID-19 pandemic and beyond. J Gastrointest Surg.

[18] Calton B, Abedini N, Fratkin M (2020). Telemedicine in the time of coronavirus. J Pain Symptom Manag.

[19] Suter P, Suter WN, Johnston D (2011). Theory-based telehealth and patient empowerment. Popul Health Manag.

[20] Bee YM (2016). A Smartphone Application to Deliver a Treat-to-Target Insulin Titration Algorithm in Insulin-Naive Patients With Type 2 Diabetes: A Pilot Randomized Controlled Trial. Diabetes Care.

[21] Core Team R. R: A language and environment for statistical computing. R Foundation for Statistical Computing. Vienna; 2020.

[22] Khunti K, Davies MJ, Kalra S (2013). Self-titration of insulin in the management of people with type 2 diabetes: a practical solution to improve management in primary care. Diabetes Obes Metab.

[23] Nathan DM (2009). Medical management of hyperglycemia in type 2 diabetes: a consensus algorithm for the initiation and adjustment of therapy: a consensus statement of the American Diabetes Association and the European Association for the Study of Diabetes. Diabetes Care.

[24] Nahum-Shani I (2012). Experimental design and primary data analysis methods for comparing adaptive interventions. Psychol Methods.

[25] Robins JM, Hernan MA, Brumback B (2000). Marginal structural models and causal inference in epidemiology.

[26] Deloitte, Global Mobile Consumer Survey 2016: The UK Cut. 20, Deloitte: London, UK.

[27] Kosorok, M. and E. Moodie, Adaptive Treatment Strategies in Practice. Adaptive Treatment Strategies in Practice.

[28] D'Agostino RB, Massaro JM, Sullivan LM (2003). Non-inferiority trials: design concepts and issues - the encounters of academic consultants in statistics. Stat Med.

[29] Ghosh P (2020). Noninferiority and equivalence tests in sequential, multiple assignment, randomized trials (SMARTs). Psychol Methods.

[30] Ghosh P (2019). How_to_use_the_Shiny_App.md.

[31] Thall PF, Wathen JK (2005). Covariate-adjusted adaptive randomization in a sarcoma trial with multi-stage treatments. Stat Med.

[32] Cheung YK, Chakraborty B, Davidson KW (2015). Sequential multiple assignment randomized trial (SMART) with adaptive randomization for quality improvement in depression treatment program. Biometrics.

