# Supplementary material for: Sequential Multiple Assignment Randomized Trial (SMART) to identify optimal sequences of telemedicine interventions for improving initiation of insulin therapy: A simulation study
Source: BMC Med Res Methodol. 2021 Sep 30;21:200. doi: 10.1186/s12874-021-01395-7 (PMC8481760; doi:10.1186/s12874-021-01395-7)
Supplement: Supplementary file 1 — Additional file 1. Details of data generation model and simulation algorithm. [file 12874_2021_1395_MOESM1_ESM.docx]

**Details of data generation model and simulation algorithm.**

| **Abbreviations** | **Definitions/Values** |
| --- | --- |
| $Y_{0}$ | HbA1c (%), at baseline (week 0) before initiation of treatment |
| $Y_{6}$ | HbA1c (%), at first follow-up (week 6) |
| $Y$ | HbA1c (%), at second follow-up (week 12), end of trial |
| ${\Delta Y}_{k}$ | Change in HbA1c at stage k, where k=1,2, (i.e. ${\Delta Y_{1}= Y}_{6}-Y_{o}; \Delta Y_{2}=Y-Y_{6}$) |
| ${Rc}_{N}$ | Receptiveness to Nurse Intervention: 1 if the patient is likely to adopt telephone conversations with Nurse as a new mode of care delivery; 0 otherwise |
| ${Rc}_{A}$ | Receptiveness to App Intervention: 1 if the patient is likely to adopt the mobile application as a new mode of care delivery; 0 otherwise |
| ${Rc}_{A+N}$ | Receptiveness to App + Nurse Intervention: 1 if the patient is likely to adopt the telephone conversations with Nurse and mobile application as a new mode of care delivery; 0 otherwise |
| $T_{k}$ | Intervention the participant is in at stage k, where k=1,2 |
| $\delta$ | Response Threshold: Value of HbA1c determining the response status of the patient to the intervention |
| $R$ | 1 if a responder to the intervention i.e. if the difference between HbA1c at baseline ($Y_{0}$) and HbA1c at first follow-up ($Y_{6}$) is less than the response threshold ($\delta$), i.e. $\Delta Y_{1}<\delta$; 0 otherwise |
| $AI_{j}$ | The j-th adaptive intervention, where j = 1,2,3,4. The adaptive interventions are embedded for SMART. For RCT, the adaptive interventions are also the separate arms. |
| $C_{A}$ | The cost of the App intervention over 6 weeks. |
| $C_{N}$ | The cost of the Nurse intervention over 6 weeks. |
| $C_{A+N}$ | The cost of the App + Nurse intervention over 6 weeks. |
| $C_{switch}$ | The cost of switching between interventions (e.g. patient receives Nurse at stage 1, then switches to App at stage 2). |

**Model Development**

Baseline HbA1c ($Y_{0}$): The HbA1c of the 63 participants from the pilot study varied from 7.8 to 13 with a mean of 9.73 and a standard deviation (SD) of 1.37. Based on this data, we will simulate the baseline HbA1c from the normal distribution with mean 9.73 and SD 1.37, and trimming at 7.8 and 13.

Receptiveness to Nurse (${Rc}_{N}$), App (${Rc}_{A}$) and App + Nurse (${Rc}_{A+N}$): Since the pilot study did not track any variables pertaining to the health behavior of the participants (except “Who will use Diabetes Pal application during treatment?”)*,* the receptiveness variable was introduced based on Deloitte’s Global Mobile Consumer Survey 2016 for UK. In the survey, Deloitte had collected data on consumer behavior for the UK’s smartphone users on a variety of topics. Of particular interest were the questions pertaining to the use of smartphones to communicate via the phone calls, and the number and the type of applications consumers installed on their phones. The data revealed that only 69% respondents made weekly standard call, down from 96% in 2012, and 51% had six or more apps installed on their phones (excluding any pre-installed apps). Further, the survey revealed the top activities carried out using an app were (in descending order of usage) Weather, Social Networking, Navigation, Email, Play games, Online banking, Read the news, Streaming music, Streaming video, and Online shopping/retail. Among these, excluding the apps for communication (e.g. social networking and email) and excluding the ones which require minimal user input (e.g. weather), majority of the apps are used to accomplish tasks with user’s inputs and are interactive in nature (the user receives an output). For example, navigation apps need the user to input destination, through which the app generates the route based on a certain set of algorithms. Similarly, the app used in the pilot study requires the users to input their current blood glucose levels, based on which an algorithm generates the amount of insulin the patient should inject.

Based on the above, we assumed that a user who made at least one weekly voice call, would be comfortable carrying out insulin titration over a phone call. Another assumption was that a user who has installed less than six apps would be more likely to use them to carry out common activities as per the survey, such as for communication or to check the weather. Accordingly, only the users who downloaded six or more apps would be comfortable accomplishing tasks using apps, tasks such as using Diabetes Pal app for insulin titration. Based on these assumptions, we defined receptiveness rate to App at 51% (${P(R}_{C_{A}}=1))$and receptiveness rate to Nurse (${P(R}_{C_{N}}=1))$, who would be helping the participants with insulin titration over a phone call, at 69%. We further assumed that the receptiveness to the app and to the nurse were independent, and that the subject is receptive to at least one of the two interventions. The combined intervention (App + Nurse) is only given at week 6 when the subject does not reach the respond threshold (as described in the following two sections). The average probability of receptiveness to the combined intervention assuming that they are initially not receptive to App or Nurse may therefore be calculated as

${P(R}_{C_{A+N}}=1)= \frac{{P(R}_{C_{A}}=0)+ {P(R}_{C_{N}}=1)-{P(R}_{C_{A}}=0){\times P(R}_{C_{N}}=1) + {P(R}_{C_{A}}=1)+ {P(R}_{C_{N}}=0)-{P(R}_{C_{A}}=1){\times P(R}_{C_{N}}=0)}{2}=\frac{0.49+ 0.69-0.49\times0.69 + 0.51+ 0.31-0.51\times0.31}{2}=0.75.$

In the pilot study, data on “Who will use Diabetes Pal application during treatment?” was collected. Patients in the app intervention could either use the app themselves, with or without external help, or an immediate family member could help carry out the titration. Among the 32 patients in the app intervention, 24 indicated at baseline that they intend to use the app by themselves, one patient indicated that they would use the app themselves but with some help, and 7 indicated that they will use the app with the help of a family member. The usage changed at the first follow-up with 25 indicating that they will use the app by themselves, and 4 with help of a family member^[[1]](#footnote-1)^. Therefore, it can be assumed that the level of comfort i.e. the receptiveness on the part of the user, whether it’s the patient themselves (more likely) or a family member guiding the patient, would impact the adherence to, and therefore the effect of the insulin treatment.

Effect of insulin therapy on HbA1c levels (${\Delta Y}_{1} and {\Delta Y}_{2})$: In order to determine the effect of insulin therapy on HbA1c levels of participants, four distributions were developed. The distributions were based on the HbA1c data collected for 63 participants in the pilot study over the period of first 12 weeks.

At the first follow-up, the mean change in HbA1c, i.e. the ${\Delta Y}_{1}$, was -0.92 with a SD of 0.71. Since the change in HbA1c was for all the participants, the separate mean reduction change for receptive and non-receptive participants were calculated. Based on the above receptiveness values, the average receptiveness comes out to be (51%+69%)/2=60%. The total mean reduction of 0.92 can therefore be written as: 0.60*(${\Delta Y}_{1}$for receptive participants)+(1-0.6)*(${\Delta Y}_{1}$for non-receptive participants)= -1.53. Assuming that the ∆Y1 for non-receptive participants is equal to 0, then ${\Delta Y}_{1}$for receptive participants= -1.53. We assume the standard deviations for receptive and non-receptive participants to be equal (SD = 0.71).

Similarly, at second follow-up the mean change, ${\Delta Y}_{2}$ was ​-0.56 with a SD of 0.77. Accordingly, the ${\Delta Y}_{2}$ for non-receptive participants is equal to 0, and ${\Delta Y}_{2}$ for receptive participants= -0.94, and standard deviations for both receptive and non-receptive participants are SD =0.77.

Selection of Response Threshold ($\delta$): While choosing the response threshold, inputs from the clinical experts, evidence from research, and data from the pilot study were all considered.

- Based on the literature and clinical inputs, insulin therapy is known to reduce the HbA1c levels in the range of 1.5 to 3.5 (conditional on baseline values) and is rapidly effective^2^.
- In the pilot study, however, the decrease of 0.92 (SD=0.71) was measured at week 6 and that of 0.56 (SD=0.77) measured at week 12 with the baseline HbA1c values equal to 9.73 (SD = 1.37). As expected, the HbA1c decrease was prominent from baseline to week 6 but less marked thereafter.

Based on the clinical/research inputs and the pilot study data, a $\delta$ of 0.5% was selected for week 6 as a measure of response for the base case of the microsimulation. $\delta$ was also varied for sensitivity analysis.

Cost Analysis:

The data below is based on the cost incurred in the pilot study and expert inputs (Table A1) . For the microsimulation, we limited our cost estimates to the cost of personnel time only (Table A2). This includes time spent providing the intervention, monitoring, and re-randomization when needed. Total cost was then converted to US dollars (exchange rate 1.267).

*Table A1. Table of costs incurred in the pilot study and expert inputs.*

| **Cost type** | | **Assumption** | **Time required per participant** | **Cost per participant** |
| --- | --- | --- | --- | --- |
| Cost #1: Recruitment (one time cost) | 1.1 Participant consultation with Research Assistant (RA) | S$50 per hour | 1 hour | S$50 |
|  | 1.2 Participant consultation with Endocrinologist | S$300 per hour | 5 minutes | S$25 |
| Cost #2: Follow-up (recurring cost) | 2.1 Follow-up with RA for all participants | S$50 per hour | 15 minutes per week | S$75 per 6 weeks |
|  | 2.2 Additional follow-up with RA for participants in App intervention | S$50 per hour | 15 minutes per week | S$75 per 6 weeks |
|  | 2.3 Additional follow-up with Nurse for patients in Nurse and App + Nurse interventions | S$5000 per 4 weeks, 40 hour weeks | 30 minutes per week | ≈S$94 per 6 weeks |
|  | 2.4 Group discussion (Endocrinologist +RA + Nurse) on all participants | (S$5000/160+S$50 +S$300)/12 for 4 weeks | 5 minutes per 4 weeks | ≈S$48 per 6 weeks |
| Cost #3: Re-assignment of non-responders (one time cost) | Consultation with RA for all participants to be re-assignment of treatment | S$50 per hour | 1 hour | S$50 |
| Cost #4: Others | 4.1 Cost of App/Tech support (for App and App + Nurse interventions) | S$5 per participant |  | S$5 |
|  | 4.2 Training for Nurses (for Nurse and App + Nurse interventions) | S$100 per nurse, one hour of training, one trained nurse can handle 80 patients |  | S$1.25 |

*Table A2. Table of intervention costs and per participant cost in the microsimulation.*

| **Panel A: Intervention Cost** | | |
| --- | --- | --- |
| Intervention | Cost Components | Cost |
| Nurse | 2.1,2.3,2.4 | $C_{N}$ = 75 + 94 + 48=S$217 |
| App | 2.1,2.2, 2.4 | $C_{A}$=75 + 75 + 48= S$198 |
| App + Nurse | 2.1,2.3, 2.4 | $C_{A+N}$ = 75 + 94 + 48= S$217 |
| Re-assignment cost | 3 | $C_{switch}$ = S$50 |
| **Panel B: Cost per participant** | | |
| Participant intervention path | Cost Components | Cost |
| Nurse at stage 1, continue with Nurse at stage 2 as a responder (N, N) | $C_{N}\times2$ | S$434  (US$ 342.54) |
| Nurse at stage 1, switch to App at stage 2 as a non-responder (N, A) | $C_{N}+C_{A}+C_{switch}$ | S$465  (US$ 367.01) |
| App at stage 1, continue with App at stage 2 as a responder (A, A) | $C_{A}\times2$ | S$396 (US$ 312.55) |
| App at stage 1, switch to Nurse at stage 2 as a non-responder (A, N) | $C_{A}+C_{N}+C_{switch}$ | S$465 (US$367.01) |
| Nurse at stage 1, switch to App + Nurse at stage 2 as a non-responder (N, A+N) | $C_{N}+C_{A+N}+C_{switch}$ | S$484 (US$382.00) |
| App at stage 1, switch to App + Nurse at stage 2 as a non-responder (A, A+N) | $C_{A}+C_{A+N}+C_{switch}$ | S$465 (US$367.01) |

**Data Generation Algorithm**

**Sequential Multiple Assignment Randomized Trial (SMART)**

1. Draw the baseline H1BAc ($Y_{0}$), from the normal distribution, with mean = 9.73 and SD =1.37, and cut-off at (7.8,13), for $n$ patients.
2. Assume equal randomization to $T_{1}$. At stage 1, randomly allocate nA=floor(n/2) patients to App, and n-nA to Nurse.
3. Generate the receptiveness for each patient, where 1 is receptive to$T_{1}$, and 0 otherwise. For patients allocated to App, draw from $Ber\left( P\left( R_{C_{A}}=1 \right) \right)$ For patients allocated to Nurse, draw from $Ber\left( P\left( R_{C_{N}}=1 \right) \right)$.
4. For patients that are receptive to their allocated $T_{1}$, draw the change in HBA1c in 6 weeks (${\Delta Y}_{1}$) from a normal distribution with mean =-1.53, SD=0.71. For non-receptive patients, draw ${\Delta Y}_{1}$ from a normal distribution with mean=0, SD=0.71.
5. Calculate the intermediate results as $Y_{6}=Y_{0}+{\Delta Y}_{1}$. If the intermediate result $Y_{6}<6$, set the value as 6.
6. For base scenario, the threshold is δ = 0.5%. Let patients with $\Delta Y_{1}<\delta$ be judged as a responder, else a non-responder.
7. Subjects that are responders stay with their initial intervention in stage 2 (i.e.$T_{2}=T_{1}$). The responders’ receptiveness to intervention at stage 2 therefore remains the same as stage 1.
8. For non-responders, the following process is repeated by subgroups categorized by $T_{1}$ and their respective receptiveness to $T_{1}$ (i.e. {$T_{1}=A, R_{C_{A}}=1$}; {$T_{1}=A, R_{C_{A}}=0$}; {$T_{1}=N, R_{C_{N}}=1$}; {$T_{1}=N, R_{C_{N}}=0$}).
   1. Let $n_{s}$ be the number of patients in the subgroups.
   2. Randomly select floor($n_{s}$/2) patients to be switched to the other intervention (i.e. if $T_{1}$ = A, then $T_{2}$ = N). Draw the receptiveness to the new intervention as detailed in step 3.
   3. The remaining ($n_{s}$− floor($n_{s}$/2) ) patients are allocated to $A+N$. Draw the receptiveness to the new intervention from $Ber\left( P\left( R_{C_{A+N}}=1 \right) \right)$.
9. For patients that are receptive to their allocated $T_{2}$., draw the change in HBA1c in the next 6 weeks ($\Delta Y_{2}$) from a normal distribution with mean = -0.94 , SD=0.77. For non-receptive patients, draw $\Delta Y_{2}$ from a normal distribution with mean =0, SD =0.77.
10. Calculate the final outcome as $Y=Y_{6}+\Delta Y_{2}$. For $Y<6$, set value as 6.
11. Calculate the cost for each patient as $cost=I\left( T_{1}=A \right)C_{A}+ I\left( T_{1}=N \right)C_{N}+ I\left( T_{2}=N \right)C_{N}+ I\left( T_{2}=A \right)C_{A}+ I\left( T_{2}=A+N \right)C_{A+N}+ I\left( T_{1}\neq T_{2} \right)C_{switch}$.
12. Simulate step 1 to 11 for B = 10000 simulations.

**Randomized Controlled Trial (RCT)**

1. Draw the baseline H1BAc ($Y_{0}$), from the normal distribution, with mean = 9.73 and SD =1.37, and cut-off at (7.8,13), for $n$ patients.
2. For RCT, patients are randomized to one of the four arms (i.e. $\mathrm{AI}_{1},\mathrm{AI}_{2}, \mathrm{AI}_{3},\mathrm{AI}_{4}$) at the start. Assuming equal randomization, randomly allocate nAI1= floor(floor(n/2)/2), nAI2 = floor(n/2) - nAI1, nAI3 = floor( (n - (nAi1 + nAi2)) /2) and nAI4 = (n - (nAI1 + nAI2)) - nAI3 patients to $\mathrm{AI}_{1},\mathrm{AI}_{2}, \mathrm{AI}_{3},\mathrm{AI}_{4}$ respectively.
3. The subsequent steps follow step 3 onwards in SMART, except there will be no re-randomization for non-responders at step 8 (i.e. All non-responders are given the intervention as specified by their allocated arm at the start).

1. The remaining 3 discontinued the intervention.

   2 Nathan, David M., et al. "Medical management of hyperglycemia in type 2 diabetes: a consensus algorithm for the initiation and adjustment of therapy: a consensus statement of the American Diabetes Association and the European Association for the Study of Diabetes." Diabetes care 32.1 (2009): 193-203. [↑](#footnote-ref-1)
